# (−)-Dimethyl 3,3′-diphenyl-2,2′-[pyridine-2,6-diylbis(carbonyl­imino)]dipropanoate

**DOI:** 10.1107/S1600536809023721

**Published:** 2009-06-27

**Authors:** Shaohong Yin, Yu Cui, Guangming Xia, Yanju Zhang, Guoxin Sun

**Affiliations:** a School of Chemistry and Chemical Engineering, University of Jinan, Jinan 250022, People’s Republic of China

## Abstract

The title compound, C_27_H_27_N_3_O_6_, a bis-amide derivative, is also a chiral amino acid ester with l-phenyl­alanine methyl ester groups as amine substituents. The pyridine ring is oriented at dihedral angles of 89.69 (3) and 62.95 (3)° with respect to the phenyl rings, while the dihedral angle between the phenyl rings is 60.76 (3)°. In the crystal structure, inter­molecular N—H⋯O hydrogen bonds link the mol­ecules into chains. One of the carbonyl O atoms and one of the meth­oxy CH_3_ groups are disordered over two positions. The O atom was refined with occupancies of 0.69 (13) and 0.31 (13), while C and H atoms were refined with occupancies of 0.69 (8) and 0.31 (8).

## Related literature

For general background, see: Darshan *et al.* (1998 ▶). For related structures, see: Amr *et al.* (1999 ▶); Moriuchi *et al.* (2006 ▶). For bond-length data, see: Allen *et al.* (1987 ▶).
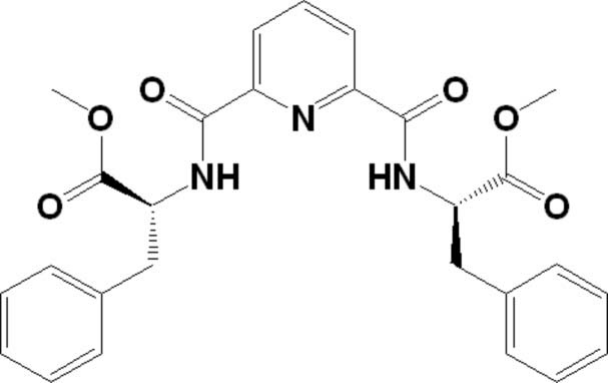


## Experimental

### 

#### Crystal data

C_27_H_27_N_3_O_6_
*M* *_r_* = 489.52
Orthorhombic, 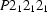

*a* = 9.1549 (11) Å
*b* = 9.9319 (12) Å
*c* = 27.83 (2) Å
*V* = 2530 (2) Å^3^
*Z* = 4
Mo *K*α radiation
μ = 0.09 mm^−1^
*T* = 293 K
0.15 × 0.10 × 0.10 mm

#### Data collection

Bruker SMART CCD area-detector diffractometer
Absorption correction: multi-scan (*SADABS*; Sheldrick, 1996 ▶) *T*_min_ = 0.986, *T*_max_ = 0.991
12674 measured reflections
2650 independent reflections
1929 reflections with *I* > 2σ(*I*)
*R*_int_ = 0.040

#### Refinement

*R*[*F* ^2^ > 2σ(*F* ^2^)] = 0.046
*wR*(*F* ^2^) = 0.134
*S* = 1.05
2650 reflections
349 parameters
H-atom parameters constrained
Δρ_max_ = 0.27 e Å^−3^
Δρ_min_ = −0.20 e Å^−3^

### 

Data collection: *SMART* (Bruker, 2001 ▶); cell refinement: *SAINT* (Bruker, 2001 ▶); data reduction: *SAINT*; program(s) used to solve structure: *SHELXS97* (Sheldrick, 2008 ▶); program(s) used to refine structure: *SHELXS97* (Sheldrick, 2008 ▶); molecular graphics: *SHELXTL* (Sheldrick, 2008 ▶); software used to prepare material for publication: *SHELXTL*.

## Supplementary Material

Crystal structure: contains datablocks global, I. DOI: 10.1107/S1600536809023721/hk2713sup1.cif

Structure factors: contains datablocks I. DOI: 10.1107/S1600536809023721/hk2713Isup2.hkl

Additional supplementary materials:  crystallographic information; 3D view; checkCIF report

## Figures and Tables

**Table 1:** Hydrogen-bond geometry (Å, °)

*D*—H⋯*A*	*D*—H	H⋯*A*	*D*⋯*A*	*D*—H⋯*A*
N2—H2⋯O1^i^	0.86	2.37	3.178 (4)	157
N3—H3⋯O1^i^	0.86	2.40	3.190 (4)	154

Symmetry code: (i) 
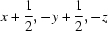
.

**Fig. 1.**
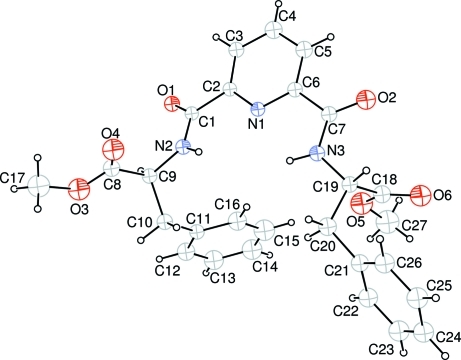
The molecular structure of the title molecule, with the atom-numbering scheme.

## supplementary crystallographic information

### Comment

The chiral bisamide derived from pyridine-2,6-dicarboxylic acid and natural amino acids adopts spontaneously relatively rigid conformation reinforced by bifurcated hydrogen bonding between NH of carboxamides at positions 2 and 6 of the pyridine nucleus and its nitrogen (Darshan *et al.*, 1998). This finding makes this kind of structures very promising for biological activities and as precursors in the syntheses of various compounds.

In the structure of the title compound (Fig 1), the bond lengths (Allen *et al.*, 1987) and angles are within normal ranges. Rings A (N1/C2-C6), B (C11-C16) and C (C21-C26) are, of course, planar and the dihedral angles between them are A/B = 89.69 (3), A/C = 62.95 (3) and B/C = 60.76 (3)°, respectively. The absolute configuration was determined by comparison with Amr *et al.* (1999) and according to the known S configuration at the C atom to which the benzyl group is attached. Both of C9 and C19 are chiral atoms in the structure. The pyridine-2,6-dicarboxamide core approximates *C*_2_ point symmetry. Such a feature seems to be common for symmetrically substituted pyridine-2,6-dicarboxamide derivatives.

In the crystal structure, intermolecular N—H···O hydrogen bonds (Table 1) link the molecules into chains, in which they may be effective in the stabilization of the structure.

### Experimental

The title compound was synthesized by a slight modification of the literature method (Moriuchi *et al.*, 2006). To a stirred mixture of L-phenylalanine methyl ester hydrochloride (129.4 mg, 0.6 mmol) in dry dichloromethane (15 ml) and triethylamine (0.21 ml, 1.5 mmol) was added dropwise 2,6-pyridyldicarbonyl dichloride (61.2 mg, 0.3 mmol) in dichloromethane (3 ml) at 273 K, and then stirred for 18 h at room temperature. The resulting mixture was diluted with dichloromethane, washed with saturated NaHCO_3_ solution and brine, and then dried over anhydrous MgSO_4_. The solvent was evaporated *in vacuo*. The title compound was isolated as a colorless solid by recrystallization from ethanol (yield; 117.5 mg, 80%; m.p. 403-404 K, enantiomeric excess >99%). Crystals suitable for X-ray analysis were obtained from the mixed solution of ethanol and diisopropyl ether by slow evaporation over a period of several days. C_27_H_27_N_3_O_6_: C 66.25, H 5.56, N 8.58%; found: C 66.11, H 5.46, N 8.64%. IR (KBr): *v* = 3398, 3333, 3028, 1745, 1678, 1523 cm^-1^.

### Refinement

The O4, C27, H27A, H27B and H27C atoms were disordered. During the refinement process, the disordered C and H atoms were refined with occupancies of 0.69 (8) and 0.31 (8), while O atom was refined with occupancies of 0.69 (13) and 0.31 (13). H atoms were positioned geometrically, with N-H = 0.86 Å (for NH) and C-H = 0.93, 0.98, 0.97 and 0.96 Å for aromatic, methine, methylene and methyl H, respectively, and constrained to ride on their parent atoms, with U_iso_(H) = xU_eq_(C,N), where x = 1.5 for methyl H and x = 1.2 for all other H atoms. In the absence of significant anomalous dispersion effects, Friedel pairs were averaged.

### Crystal data

**Table:**

C_27_H_27_N_3_O_6_	*F*(000) = 1032
*M**_r_* = 489.52	*D*_x_ = 1.285 Mg m^−^^3^
Orthorhombic, *P*2_1_2_1_2_1_	Mo *K*α radiation, λ = 0.71073 Å
Hall symbol: P 2ac 2ab	Cell parameters from 2406 reflections
*a* = 9.1549 (11) Å	θ = 2.3–20.1°
*b* = 9.9319 (12) Å	µ = 0.09 mm^−^^1^
*c* = 27.83 (2) Å	*T* = 294 K
*V* = 2530 (2) Å^3^	Prism, colorless
*Z* = 4	0.15 × 0.10 × 0.10 mm

### Data collection

**Table:**

Bruker SMART CCD area-detector diffractometer	2650 independent reflections
Radiation source: fine-focus sealed tube	1929 reflections with *I* > 2σ(*I*)
graphite	*R*_int_ = 0.040
φ and ω scans	θ_max_ = 25.4°, θ_min_ = 2.2°
Absorption correction: multi-scan (*SADABS*; Sheldrick, 1996)	*h* = −10→11
*T*_min_ = 0.986, *T*_max_ = 0.991	*k* = −11→10
12674 measured reflections	*l* = −33→32

### Refinement

**Table:**

Refinement on *F*^2^	Primary atom site location: structure-invariant direct methods
Least-squares matrix: full	Secondary atom site location: difference Fourier map
*R*[*F*^2^ > 2σ(*F*^2^)] = 0.046	Hydrogen site location: inferred from neighbouring sites
*wR*(*F*^2^) = 0.134	H-atom parameters constrained
*S* = 1.05	*w* = 1/[σ^2^(*F*_o_^2^) + (0.1*P*)^2^ + 0.1227*P*] where *P* = (*F*_o_^2^ + 2*F*_c_^2^)/3
2650 reflections	(Δ/σ)_max_ = 0.001
349 parameters	Δρ_max_ = 0.27 e Å^−^^3^
0 restraints	Δρ_min_ = −0.20 e Å^−^^3^

### Special details

**Table:**

Geometry. All e.s.d.'s (except the e.s.d. in the dihedral angle between two l.s. planes) are estimated using the full covariance matrix. The cell e.s.d.'s are taken into account individually in the estimation of e.s.d.'s in distances, angles and torsion angles; correlations between e.s.d.'s in cell parameters are only used when they are defined by crystal symmetry. An approximate (isotropic) treatment of cell e.s.d.'s is used for estimating e.s.d.'s involving l.s. planes.
Refinement. Refinement of *F*^2^ against ALL reflections. The weighted *R*-factor *wR* and goodness of fit *S* are based on *F*^2^, conventional *R*-factors *R* are based on *F*, with *F* set to zero for negative *F*^2^. The threshold expression of *F*^2^ > σ(*F*^2^) is used only for calculating *R*-factors(gt) *etc*. and is not relevant to the choice of reflections for refinement. *R*-factors based on *F*^2^ are statistically about twice as large as those based on *F*, and *R*- factors based on ALL data will be even larger.

### Fractional atomic coordinates and isotropic or equivalent isotropic displacement parameters (Å^2^)

**Table:**

	*x*	*y*	*z*	*U*_iso_*/*U*_eq_	Occ. (<1)
O1	0.2953 (3)	0.1251 (2)	−0.02271 (8)	0.0591 (6)	
O2	0.6669 (3)	0.1803 (3)	0.19298 (10)	0.0826 (9)	
O3	0.5457 (4)	0.3451 (4)	−0.13664 (12)	0.1087 (12)	
O4	0.687 (6)	0.228 (3)	−0.0887 (8)	0.101 (8)	0.69 (13)
O4'	0.614 (14)	0.190 (8)	−0.0963 (17)	0.101 (18)	0.31 (13)
O5	0.5417 (3)	0.5103 (4)	0.17725 (10)	0.1148 (13)	
O6	0.6902 (3)	0.4951 (4)	0.23889 (10)	0.0983 (11)	
N1	0.5319 (3)	0.1704 (3)	0.07475 (9)	0.0469 (6)	
N2	0.4923 (3)	0.2592 (3)	−0.01478 (9)	0.0536 (7)	
H2	0.5678	0.2767	0.0025	0.064*	
N3	0.7013 (3)	0.3214 (3)	0.13144 (10)	0.0529 (7)	
H3	0.6951	0.3330	0.1009	0.063*	
C1	0.4044 (3)	0.1610 (3)	−0.00050 (11)	0.0458 (8)	
C2	0.4433 (3)	0.0987 (3)	0.04660 (11)	0.0471 (8)	
C3	0.3850 (4)	−0.0227 (3)	0.06022 (14)	0.0580 (9)	
H3A	0.3274	−0.0720	0.0391	0.070*	
C4	0.4132 (4)	−0.0703 (4)	0.10552 (16)	0.0751 (12)	
H4	0.3743	−0.1520	0.1156	0.090*	
C5	0.4999 (4)	0.0044 (4)	0.13576 (14)	0.0686 (10)	
H5	0.5191	−0.0246	0.1669	0.082*	
C6	0.5579 (4)	0.1239 (3)	0.11873 (12)	0.0522 (8)	
C7	0.6480 (4)	0.2102 (4)	0.15113 (13)	0.0556 (9)	
C8	0.5704 (5)	0.2909 (4)	−0.09640 (15)	0.0655 (11)	
C9	0.4695 (4)	0.3387 (3)	−0.05746 (12)	0.0535 (8)	
H9	0.3687	0.3254	−0.0683	0.064*	
C10	0.4920 (4)	0.4888 (4)	−0.04709 (13)	0.0603 (9)	
H10A	0.5885	0.5015	−0.0334	0.072*	
H10B	0.4885	0.5379	−0.0772	0.072*	
C11	0.3809 (4)	0.5473 (3)	−0.01345 (13)	0.0556 (9)	
C12	0.2661 (5)	0.6211 (5)	−0.03059 (18)	0.0831 (13)	
H12	0.2566	0.6349	−0.0635	0.100*	
C13	0.1648 (5)	0.6748 (5)	0.0002 (3)	0.1036 (18)	
H13	0.0881	0.7256	−0.0121	0.124*	
C14	0.1745 (7)	0.6555 (6)	0.0479 (3)	0.111 (2)	
H14	0.1050	0.6917	0.0685	0.133*	
C15	0.2870 (9)	0.5825 (5)	0.0652 (2)	0.116 (2)	
H15	0.2950	0.5683	0.0981	0.139*	
C16	0.3896 (6)	0.5291 (5)	0.03496 (16)	0.0873 (14)	
H16	0.4666	0.4796	0.0477	0.105*	
C17	0.6428 (7)	0.3105 (6)	−0.17656 (16)	0.1132 (19)	
H17A	0.6814	0.2216	−0.1717	0.170*	
H17B	0.7218	0.3740	−0.1780	0.170*	
H17C	0.5891	0.3131	−0.2062	0.170*	
C18	0.6648 (4)	0.4771 (5)	0.19758 (15)	0.0697 (11)	
C19	0.7696 (4)	0.4234 (4)	0.16093 (12)	0.0551 (9)	
H19	0.8507	0.3809	0.1782	0.066*	
C20	0.8326 (5)	0.5343 (4)	0.12987 (13)	0.0701 (11)	
H20A	0.9090	0.4966	0.1098	0.084*	
H20B	0.7565	0.5681	0.1088	0.084*	
C21	0.8940 (4)	0.6484 (4)	0.15781 (13)	0.0634 (10)	
C22	0.8182 (6)	0.7669 (4)	0.16388 (15)	0.0797 (12)	
H22	0.7274	0.7766	0.1493	0.096*	
C23	0.8722 (6)	0.8708 (5)	0.19077 (17)	0.0904 (14)	
H23	0.8181	0.9494	0.1945	0.108*	
C24	1.0036 (6)	0.8593 (5)	0.21183 (15)	0.0843 (13)	
H24	1.0415	0.9304	0.2297	0.101*	
C25	1.0805 (5)	0.7442 (5)	0.20703 (16)	0.0881 (14)	
H25	1.1707	0.7355	0.2221	0.106*	
C26	1.0258 (5)	0.6390 (5)	0.17983 (16)	0.0797 (12)	
H26	1.0804	0.5605	0.1766	0.096*	
C27	0.440 (2)	0.592 (5)	0.2070 (10)	0.135 (10)	0.69 (8)
H27A	0.3787	0.5336	0.2256	0.202*	0.69 (8)
H27B	0.3802	0.6469	0.1864	0.202*	0.69 (8)
H27C	0.4951	0.6491	0.2281	0.202*	0.69 (8)
C27'	0.425 (4)	0.506 (11)	0.220 (2)	0.135 (17)	0.31 (8)
H27D	0.4288	0.4196	0.2350	0.202*	0.31 (8)
H27E	0.3294	0.5207	0.2067	0.202*	0.31 (8)
H27F	0.4475	0.5751	0.2425	0.202*	0.31 (8)

### Atomic displacement parameters (Å^2^)

**Table:**

	*U*^11^	*U*^22^	*U*^33^	*U*^12^	*U*^13^	*U*^23^
O1	0.0541 (13)	0.0687 (15)	0.0544 (14)	−0.0128 (12)	−0.0117 (12)	−0.0002 (12)
O2	0.098 (2)	0.098 (2)	0.0523 (17)	−0.0263 (19)	−0.0149 (16)	0.0129 (15)
O3	0.130 (3)	0.128 (3)	0.068 (2)	0.034 (2)	0.019 (2)	0.033 (2)
O4	0.099 (18)	0.123 (9)	0.080 (5)	0.045 (11)	0.009 (7)	0.010 (5)
O4'	0.10 (4)	0.123 (19)	0.080 (11)	0.05 (2)	0.009 (16)	0.010 (11)
O5	0.077 (2)	0.192 (4)	0.076 (2)	0.046 (2)	−0.0100 (17)	−0.047 (2)
O6	0.099 (2)	0.141 (3)	0.0547 (17)	0.011 (2)	−0.0081 (17)	−0.0303 (18)
N1	0.0451 (14)	0.0508 (14)	0.0448 (16)	−0.0010 (13)	0.0018 (12)	0.0015 (12)
N2	0.0489 (14)	0.0586 (16)	0.0533 (17)	−0.0097 (14)	−0.0105 (13)	0.0049 (14)
N3	0.0620 (17)	0.0578 (17)	0.0389 (15)	−0.0040 (15)	−0.0052 (14)	−0.0047 (13)
C1	0.0412 (16)	0.0472 (17)	0.0491 (19)	0.0007 (15)	0.0007 (15)	−0.0056 (15)
C2	0.0454 (17)	0.0494 (18)	0.0466 (19)	0.0011 (15)	−0.0019 (15)	−0.0021 (15)
C3	0.055 (2)	0.051 (2)	0.068 (2)	−0.0062 (16)	−0.0075 (18)	0.0033 (18)
C4	0.076 (3)	0.062 (2)	0.088 (3)	−0.015 (2)	−0.011 (2)	0.020 (2)
C5	0.076 (2)	0.068 (2)	0.062 (2)	−0.009 (2)	−0.008 (2)	0.0157 (19)
C6	0.0499 (18)	0.0537 (19)	0.053 (2)	−0.0023 (16)	−0.0027 (16)	0.0035 (16)
C7	0.058 (2)	0.066 (2)	0.043 (2)	−0.0020 (18)	−0.0017 (17)	0.0023 (17)
C8	0.085 (3)	0.054 (2)	0.058 (3)	0.004 (2)	−0.002 (2)	0.0019 (19)
C9	0.0523 (19)	0.0548 (19)	0.053 (2)	−0.0071 (16)	−0.0062 (16)	0.0070 (17)
C10	0.059 (2)	0.059 (2)	0.063 (2)	−0.0070 (19)	−0.0017 (18)	0.0081 (18)
C11	0.058 (2)	0.0498 (19)	0.059 (2)	−0.0076 (16)	−0.0035 (18)	0.0008 (17)
C12	0.079 (3)	0.087 (3)	0.083 (3)	0.016 (3)	−0.014 (2)	−0.019 (2)
C13	0.067 (3)	0.096 (4)	0.147 (5)	0.013 (3)	−0.010 (3)	−0.043 (4)
C14	0.113 (4)	0.079 (4)	0.140 (6)	−0.025 (3)	0.051 (4)	−0.034 (4)
C15	0.189 (7)	0.075 (3)	0.083 (4)	−0.008 (4)	0.043 (4)	−0.005 (3)
C16	0.122 (4)	0.075 (3)	0.065 (3)	0.014 (3)	0.000 (3)	0.000 (2)
C17	0.162 (5)	0.111 (4)	0.067 (3)	−0.006 (4)	0.029 (3)	0.008 (3)
C18	0.058 (2)	0.096 (3)	0.055 (2)	0.002 (2)	−0.010 (2)	−0.011 (2)
C19	0.058 (2)	0.063 (2)	0.0443 (19)	−0.0005 (17)	−0.0054 (16)	−0.0081 (16)
C20	0.084 (3)	0.076 (3)	0.050 (2)	−0.016 (2)	−0.001 (2)	−0.0069 (19)
C21	0.074 (2)	0.068 (2)	0.048 (2)	−0.012 (2)	−0.0036 (18)	−0.0025 (18)
C22	0.095 (3)	0.070 (3)	0.074 (3)	−0.003 (2)	−0.027 (3)	0.003 (2)
C23	0.119 (4)	0.064 (3)	0.088 (3)	−0.004 (3)	−0.018 (3)	0.001 (2)
C24	0.108 (4)	0.068 (3)	0.076 (3)	−0.017 (3)	−0.014 (3)	−0.011 (2)
C25	0.077 (3)	0.099 (3)	0.088 (3)	−0.011 (3)	−0.018 (3)	−0.016 (3)
C26	0.070 (3)	0.081 (3)	0.087 (3)	−0.002 (2)	−0.003 (2)	−0.017 (2)
C27	0.091 (7)	0.21 (3)	0.107 (10)	0.071 (13)	0.013 (7)	−0.021 (13)
C27'	0.091 (15)	0.21 (5)	0.11 (2)	0.07 (3)	0.013 (15)	−0.02 (3)

### Geometric parameters (Å, °)

**Table:**

O1—C1	1.228 (4)	C12—C13	1.371 (7)
O2—C7	1.214 (4)	C12—H12	0.9300
O3—C8	1.263 (5)	C13—C14	1.343 (8)
O3—C17	1.464 (6)	C13—H13	0.9300
O4—C8	1.25 (4)	C14—C15	1.348 (8)
O4'—C8	1.08 (3)	C14—H14	0.9300
O5—C18	1.304 (5)	C15—C16	1.367 (7)
O5—C27	1.488 (18)	C15—H15	0.9300
O5—C27'	1.59 (5)	C16—H16	0.9300
O6—C18	1.186 (4)	C17—H17A	0.9600
N1—C2	1.333 (4)	C17—H17B	0.9600
N1—C6	1.330 (4)	C17—H17C	0.9600
N2—C1	1.325 (4)	C18—C19	1.498 (5)
N2—C9	1.441 (4)	C19—C20	1.514 (5)
N2—H2	0.8600	C19—H19	0.9800
N3—C7	1.327 (5)	C20—C21	1.485 (5)
N3—C19	1.445 (4)	C20—H20A	0.9700
N3—H3	0.8600	C20—H20B	0.9700
C1—C2	1.493 (5)	C21—C26	1.357 (6)
C2—C3	1.373 (5)	C21—C22	1.377 (6)
C3—C4	1.371 (5)	C22—C23	1.367 (6)
C3—H3A	0.9300	C22—H22	0.9300
C4—C5	1.374 (5)	C23—C24	1.343 (7)
C4—H4	0.9300	C23—H23	0.9300
C5—C6	1.384 (5)	C24—C25	1.349 (7)
C5—H5	0.9300	C24—H24	0.9300
C6—C7	1.492 (5)	C25—C26	1.384 (6)
C8—C9	1.501 (6)	C25—H25	0.9300
C9—C10	1.532 (5)	C26—H26	0.9300
C9—H9	0.9800	C27—H27A	0.9600
C10—C11	1.499 (5)	C27—H27B	0.9600
C10—H10A	0.9700	C27—H27C	0.9600
C10—H10B	0.9700	C27'—H27D	0.9600
C11—C16	1.361 (5)	C27'—H27E	0.9600
C11—C12	1.368 (5)	C27'—H27F	0.9600
			
C8—O3—C17	117.7 (4)	C13—C14—C15	118.7 (5)
C18—O5—C27	116.0 (9)	C13—C14—H14	120.7
C18—O5—C27'	105 (2)	C15—C14—H14	120.7
C6—N1—C2	117.7 (3)	C14—C15—C16	120.9 (5)
C1—N2—C9	124.2 (3)	C14—C15—H15	119.5
C1—N2—H2	117.9	C16—C15—H15	119.5
C9—N2—H2	117.9	C11—C16—C15	121.1 (5)
C7—N3—C19	120.5 (3)	C11—C16—H16	119.5
C7—N3—H3	119.7	C15—C16—H16	119.5
C19—N3—H3	119.7	O3—C17—H17A	109.5
O1—C1—N2	123.9 (3)	O3—C17—H17B	109.5
O1—C1—C2	121.1 (3)	H17A—C17—H17B	109.5
N2—C1—C2	115.0 (3)	O3—C17—H17C	109.5
N1—C2—C3	122.9 (3)	H17A—C17—H17C	109.5
N1—C2—C1	116.1 (3)	H17B—C17—H17C	109.5
C3—C2—C1	120.9 (3)	O6—C18—O5	123.5 (4)
C4—C3—C2	118.9 (3)	O6—C18—C19	126.0 (4)
C4—C3—H3A	120.5	O5—C18—C19	110.4 (3)
C2—C3—H3A	120.5	N3—C19—C18	111.1 (3)
C3—C4—C5	119.1 (4)	N3—C19—C20	110.5 (3)
C3—C4—H4	120.5	C18—C19—C20	111.9 (3)
C5—C4—H4	120.5	N3—C19—H19	107.7
C4—C5—C6	118.3 (4)	C18—C19—H19	107.7
C4—C5—H5	120.8	C20—C19—H19	107.7
C6—C5—H5	120.8	C21—C20—C19	113.6 (3)
N1—C6—C5	123.0 (3)	C21—C20—H20A	108.8
N1—C6—C7	117.1 (3)	C19—C20—H20A	108.8
C5—C6—C7	119.8 (3)	C21—C20—H20B	108.8
O2—C7—N3	123.2 (3)	C19—C20—H20B	108.8
O2—C7—C6	121.2 (3)	H20A—C20—H20B	107.7
N3—C7—C6	115.6 (3)	C26—C21—C22	116.8 (4)
O4'—C8—O3	118 (3)	C26—C21—C20	121.4 (4)
O4—C8—O3	121.1 (7)	C22—C21—C20	121.7 (4)
O4'—C8—C9	121 (3)	C23—C22—C21	122.0 (4)
O4—C8—C9	123.9 (7)	C23—C22—H22	119.0
O3—C8—C9	113.3 (4)	C21—C22—H22	119.0
N2—C9—C8	109.5 (3)	C24—C23—C22	119.9 (5)
N2—C9—C10	111.0 (3)	C24—C23—H23	120.0
C8—C9—C10	111.2 (3)	C22—C23—H23	120.0
N2—C9—H9	108.4	C23—C24—C25	119.7 (4)
C8—C9—H9	108.4	C23—C24—H24	120.1
C10—C9—H9	108.4	C25—C24—H24	120.1
C11—C10—C9	113.8 (3)	C24—C25—C26	120.4 (4)
C11—C10—H10A	108.8	C24—C25—H25	119.8
C9—C10—H10A	108.8	C26—C25—H25	119.8
C11—C10—H10B	108.8	C21—C26—C25	121.1 (4)
C9—C10—H10B	108.8	C21—C26—H26	119.4
H10A—C10—H10B	107.7	C25—C26—H26	119.4
C16—C11—C12	117.5 (4)	O5—C27—H27A	109.5
C16—C11—C10	121.8 (4)	O5—C27—H27B	109.5
C12—C11—C10	120.7 (4)	O5—C27—H27C	109.5
C11—C12—C13	120.7 (5)	O5—C27'—H27D	109.5
C11—C12—H12	119.7	O5—C27'—H27E	109.5
C13—C12—H12	119.7	H27D—C27'—H27E	111.0
C14—C13—C12	121.2 (5)	O5—C27'—H27F	109.5
C14—C13—H13	119.4	H27D—C27'—H27F	109.5
C12—C13—H13	119.4	H27E—C27'—H27F	109.5
			
C9—N2—C1—O1	1.6 (5)	C8—C9—C10—C11	172.0 (3)
C9—N2—C1—C2	−175.8 (3)	C9—C10—C11—C16	79.1 (5)
C6—N1—C2—C3	−3.4 (5)	C9—C10—C11—C12	−100.7 (4)
C6—N1—C2—C1	173.9 (3)	C16—C11—C12—C13	0.4 (6)
O1—C1—C2—N1	−159.1 (3)	C10—C11—C12—C13	−179.8 (4)
N2—C1—C2—N1	18.4 (4)	C11—C12—C13—C14	−0.7 (8)
O1—C1—C2—C3	18.2 (5)	C12—C13—C14—C15	0.5 (8)
N2—C1—C2—C3	−164.3 (3)	C13—C14—C15—C16	0.1 (8)
N1—C2—C3—C4	3.0 (5)	C12—C11—C16—C15	0.2 (7)
C1—C2—C3—C4	−174.1 (3)	C10—C11—C16—C15	−179.7 (4)
C2—C3—C4—C5	−0.6 (6)	C14—C15—C16—C11	−0.4 (8)
C3—C4—C5—C6	−1.3 (6)	C27—O5—C18—O6	9(3)
C2—N1—C6—C5	1.3 (5)	C27'—O5—C18—O6	−27 (4)
C2—N1—C6—C7	−175.5 (3)	C27—O5—C18—C19	−167 (2)
C4—C5—C6—N1	1.0 (6)	C27'—O5—C18—C19	157 (4)
C4—C5—C6—C7	177.7 (3)	C7—N3—C19—C18	−60.0 (4)
C19—N3—C7—O2	−8.3 (6)	C7—N3—C19—C20	175.2 (3)
C19—N3—C7—C6	169.9 (3)	O6—C18—C19—N3	134.5 (5)
N1—C6—C7—O2	174.0 (3)	O5—C18—C19—N3	−49.4 (5)
C5—C6—C7—O2	−3.0 (6)	O6—C18—C19—C20	−101.5 (5)
N1—C6—C7—N3	−4.3 (5)	O5—C18—C19—C20	74.6 (5)
C5—C6—C7—N3	178.8 (3)	N3—C19—C20—C21	175.3 (3)
C17—O3—C8—O4'	−33 (9)	C18—C19—C20—C21	50.9 (5)
C17—O3—C8—O4	12 (3)	C19—C20—C21—C26	76.9 (5)
C17—O3—C8—C9	177.0 (4)	C19—C20—C21—C22	−101.0 (5)
C1—N2—C9—C8	−102.6 (4)	C26—C21—C22—C23	0.0 (7)
C1—N2—C9—C10	134.3 (3)	C20—C21—C22—C23	178.0 (4)
O4'—C8—C9—N2	24 (9)	C21—C22—C23—C24	0.6 (8)
O4—C8—C9—N2	−23 (3)	C22—C23—C24—C25	−1.2 (7)
O3—C8—C9—N2	172.4 (3)	C23—C24—C25—C26	1.2 (8)
O4'—C8—C9—C10	147 (9)	C22—C21—C26—C25	0.0 (6)
O4—C8—C9—C10	100 (3)	C20—C21—C26—C25	−178.0 (4)
O3—C8—C9—C10	−64.7 (5)	C24—C25—C26—C21	−0.6 (7)
N2—C9—C10—C11	−65.9 (4)		

### Hydrogen-bond geometry (Å, °)

**Table:**

*D*—H···*A*	*D*—H	H···*A*	*D*···*A*	*D*—H···*A*
N2—H2···O1^i^	0.86	2.37	3.178 (4)	157
N3—H3···O1^i^	0.86	2.40	3.190 (4)	154

Symmetry codes: (i) *x*+1/2, −*y*+1/2, −*z*.
